# Anti-Inflammatory Effects of Essential Oils from the Peels of Citrus Cultivars

**DOI:** 10.3390/pharmaceutics15061595

**Published:** 2023-05-25

**Authors:** Jiyoon Yang, Su-Yeon Lee, Soo-Kyeong Jang, Ki-Joong Kim, Mi-Jin Park

**Affiliations:** 1 Forest Industrial Materials Division, Forest Products and Industry Department, National Institute of Forest Science, Seoul 02455, Republic of Korea; dldh89@korea.kr (J.Y.);; 2 Division of Life Sciences, Korea University, Seoul 02841, Republic of Korea

**Keywords:** citrus, essential oil, terpenes, natural compound, anti-inflammatory, bioactivity, active compound, in vitro screening

## Abstract

Citrus cultivars have remarkable health benefits, but only the anti-inflammatory activities of the major varieties have been studied. This study investigated the anti-inflammatory effects of various citrus cultivars and their active anti-inflammatory components. The essential oils of 21 citrus peels were extracted via hydrodistillation using a Clevenger-type apparatus, and the chemical compositions of the essential oils were analyzed. D-Limonene was the most abundant constituent. To evaluate the anti-inflammatory effects of the citrus cultivars, the gene expression levels of an inflammatory mediator and proinflammatory cytokines were investigated. Among the 21 essential oils, those extracted from *C. japonica* and *C. maxima* exhibited superior anti-inflammatory activities, being able to inhibit the expression of the inflammatory mediators and proinflammatory cytokines in lipopolysaccharide-stimulated RAW 264.7 cells. The essential oils of *C. japonica* and *C. maxima* were distinguished into seven distinct constituents, α-pinene, myrcene, D-limonene, β-ocimene, linalool, linalool oxide, and α-terpineol, compared with other essential oils. The anti-inflammatory activities of the seven single compounds significantly inhibited the levels of inflammation-related factors. In particular, α-terpineol exhibited a superior anti-inflammatory effect. This study showed that the essential oils from *C. japonica* and *C. maxima* exhibit high anti-inflammatory activity. In addition, α-terpineol is an active anti-inflammatory compound that contributes to inflammatory responses.

## 1. Introduction

Inflammation is a primary defense mechanism of immune systems triggered to protect organisms from tissue-damaging stimuli [1]. Macrophages play an important role in inflammatory responses against external stimuli [2]. LPS-stimulated macrophages increase the production of proinflammatory cytokines, including *tumor necrosis factor-α* (*TNF-α*), *interleukin-1β* (*IL-1β*), and *interleukin-6* (*IL-6*) and synthesizes *inducible nitric oxide synthase* (*iNOS*) and *cyclooxygenase-2* (*COX-2*) to produce associated inflammatory mediators, such as nitric oxide (NO) and prostaglandin E_2_ (PGE_2_) [3]. NO formation kills bacteria and eliminates tumors. However, NO produced by iNOS in an inflammatory state acts as a vasodilator, promoting vascular permeability and edema, and it intensifies inflammation by promoting the biosynthesis of the inflammatory mediators [4,5]. PGE_2_, another inflammatory mediator, plays a crucial role in inhibiting the production of proinflammatory cytokines (*TNF-α*, *IL-1β*, *IL-8*, etc.) in macrophages [6]. Therefore, anti-inflammatory effects can be induced by suppressing the production of several inflammatory mediators, such as NO, PGE_2_, *TNF-α*, *IL-1β*, *IL-6*, *COX-2*, and *iNOS*, during inflammatory responses.

Steroids, antihistamines, and immunosuppressants are anti-inflammatory agents [7,8]. They relieve inflammatory symptoms in the short term, but their long-term use affects immunosuppression. In addition, there is a high risk of allergy recurrence if the medication is stopped. Therefore, there is a need for more safe and effective alternative therapies. Essential oil consists of volatile complex compounds with a strong odor. It is formed by secondary metabolites in aromatic plants [9]. It contains 20–60 components at various concentrations [10]. It consists of monoterpenes, sesquiterpenes, and phenylpropane, which comprise various functional groups, including alkanes, alcohols, aldehydes, ketones, esters, and acids [11]. These functional groups have various pharmacological properties; thus, essential oil exhibits various biological activities [12]. Among plants with pharmacological properties, *Pinus densiflora*, *Pinus koraiensis*, *Cinnamomum subavenium*, and *Chamaecyparis obtusa* exhibit good anti-inflammatory properties [13,14,15,16]. They inhibit proinflammatory cytokine expression via the nuclear factor-κB (NF-κB) pathway [17].

Citrus is a popular fruit grown commercially worldwide. It has a high economic value and nutritional benefits [18]. Citrus fruits contain physiological compounds, such as vitamins, flavonoids, phenolic compounds, and pectin. These constituents of citrus reduce cough, sore throat, stomach ailments, headache, hypertension, and hyperlipidemia [19,20]. Furthermore, essential oils from citrus cultivars exhibit various bioactivities, such as antioxidant, antibacterial, and antidepressant effects [21,22,23]. These activities of essential oils are affected by essential oil components. Essential oil components differ depending on geographical zones and the parts from which they were extracted [24]. Essential oils were extracted from the leaves and pericarps of 17 citrus fruits grown in Brazil as well as France and analyzed for their composition. The pericarp oils were richer in limonene in Brazil than in France. Meanwhile, neral and geranical were present in a higher proportion in the France cultivars.

The demand for citrus fruits has been increasing owing to the increasing interest in healthy lifestyles. Therefore, various hybrid species of citrus cultivars have been developed. The essential oils extracted from these citrus cultivars have been reported to have anti-inflammatory effects. In previous studies, *C. obovoides* and *C. natsudaidai* reduced *P. acnes*-induced secretion of *IL-8* and *TNF-α* in THP-1 cells, indicating anti-inflammatory effects [25]. In another study, the anti-inflammatory effect of essential oil from *C. unshiu* was described [26]. The essential oil was shown to suppress the production of inflammatory cytokines, including *IL-1β*, *TNF-α*, and *IL-6*, in LPS-activated RAW 264.7 macrophages. Based on these results, essential oil from *C. unshiu* is considered a potential anti-inflammatory candidate. The essential oil from *Citrus medica* L. var. *sarcodactylis* was investigated for anti-inflammatory activities by Kim et al. [27]. The essential oil inhibited LPS-stimulated inflammation by blocking the JNK, NF-κB, and ERK pathways in macrophages and demonstrated that *C. medica* possesses anti-inflammatory properties. Although various citrus cultivars grow in Korea, research on their anti-inflammatory activity has been limited to only a few species.

Therefore, this study aimed to evaluate the anti-inflammatory activity of various citrus cultivars in Korea. The study was conducted on 21 hybrid cultivars from *C. reticulata*, *C. maxima*, *C. medica*, and *C. japonica*. Furthermore, the active constituents of the essential oils with superior anti-inflammatory activity were identified.

## 2. Materials and Methods

### 2.1. Chemicals

α-Pinene (Cat #. 80604, ≥97.0%), limonene (Cat #. 62128-5ML, analytical standard), linalool (Cat #. 51782, >99.0%), linalool oxide (Cat #. 62141, ≥97.0%), and myrcene (Cat #. 64643, >90.0%) were purchased from Fluka (Buchs, Switzerland). α-Terpineol (Cat #. sc-233785, ≥97.0%) was obtained from Santa Cruz Biotechnology (Dallas, TX, USA). Moreover, β-Ocimene (Cat #. 23466, ≥90%) was purchased from Cayman Chemical (Ann Arbor, MI, USA).

### 2.2. Plant Material

The 21 citrus cultivars used in this study were collected on Jeju Island in December 2019. The identification of citrus cultivars was confirmed by Professor Ki-Joong Kim of Korea University. The voucher specimens were deposited in the National Institute of Forest Science herbarium (Table 1).

### 2.3. Essential Oil Extraction

The peels (1.0 kg) of the citrus cultivars were subjected to hydrodistillation using a Clevenger-type apparatus. The samples were distilled at 105 ± 2 °C until no more essential oil was obtained. The essential oils were collected, dried under anhydrous sodium sulfate, and stored in sealed vials in the dark at 4 °C until use.

### 2.4. Gas Chromatography–Mass Spectrometry Analysis

The chemical composition of the extracted essential oils was analyzed using a Trace 1310/ISQ-LT gas chromatographer (GC) equipped with a flame ionization detector and a VF-5MS capillary column (60 m × 0.25 mm × 0.25 μm). The injector temperature was set to 250 °C, and the flow rate of the helium carrier gas was 1.0 mL/min. The oven temperature was maintained at 50 °C for 5 min and then increased to 65 °C at a rate of 10 °C/min, after which it was increased to 210 °C (10 min) at 5 °C/min and finally to 325 °C (10 min) at 20 °C/min. The mass spectroscopy (MS) parameters were as follows: electron ionization mode: 70 eV; ion source temperature: 270 °C; and mass spectra range: 35–550 amu.

The constituents of the oil were identified by comparing their mass spectra with those reported in the NIST library. Compound identification was based on a comparison of relative retention times with those of an *n*-alkane mixture (C_8_–C_30,_ Sigma-Aldrich, St. Louis, MI, USA).

### 2.5. Cell Culture

The murine macrophage cell line, RAW 264.7, was obtained from Korea Cell Line Bank (Seoul, Republic of Korea). RAW 264.7 cells (KCLB No. 40071) were cultured in Dulbecco’s modified Eagle’s medium (Gibco, Carlsbad, CA, USA) supplemented with 10% fetal bovine serum (Gibco), 1% penicillin–streptomycin (Gibco), and 0.4 μL/mL Plasmocin™ (pH 7.4; Invivogen, San Diego, CA, USA) at 37 °C in a 5% CO_2_ incubator.

### 2.6. Cell Cytotoxic Activity

The cell cytotoxic activity of the essential oil on RAW 264.7 cells was evaluated using a cell counting kit (CCK-8, DoGenBio, Seoul, Republic of Korea). RAW 264.7 cells were seeded in 96-well plates at a density of 1 × 10^4^ cells/well and incubated at 37 °C for 24 h. The cells were treated with essential oils and single compounds (10^−5^–10^−7^%) for 24 h. After treatment, CCK-8 solution was added to each well for 1 h. The cell density was measured at 450 nm using a microplate reader (Epoch, Winooski, VT, USA). Subsequently, the percentage of cell viability was calculated using the following equation:

$$\%\;of\;cell\;viability=\frac{the\;absorbance\;of\;treated\;samples}{the\;absorbance\;of\;the\;control}\times 100\;(\%)$$

### 2.7. Determination of NO Production

To confirm the anti-inflammatory effect of the samples on RAW 264.7 cells, the presence of nitrogen dioxide (NO_2_) in the culture supernatant, as an indicator of NO production, was examined using a Griess reagent system (Invitrogen™, Waltham, MA, USA). RAW 264.7 cells were cultivated at 1 × 10^5^ cells/well in 96-well plates and then pretreated with 1 μg/mL LPS (Sigma-Aldrich). After 1 h of incubation, the cells were cultivated with dexamethasone (Sigma), the essential oils, or single compounds at 37 °C for 24 h. The supernatants (100 μL) were collected after 24 h of incubation and mixed with an equal volume of the Griess reagent, and then incubated at room temperature for 30 min. The NO concentration was determined at 548 nm in a microplate reader. Fresh culture medium was used as a blank. The quantity of nitrite was determined from the sodium nitrite standard curve.

### 2.8. Measurement of Proinflammatory Cytokine Production

RAW 264.7 cells were cultured at 1 × 10^6^ cells/well in 6-well plates. The cells were incubated in the presence of 1 μg/mL LPS for 1 h. Thereafter, they were treated with 100 nM dexamethasone, the extracted essential oils, or the single compounds for 24 h. Total RNA was isolated from macrophage cells using the standard Trizol method and detected using a Nanodrop spectrophotometer (Thermo scientific, Waltham, MA, USA). Total RNA was reverse-transcribed to cDNA using Moloney murine leukemia virus (M-MLV) reverse transcriptase (Invitrogen). For quantitative SYBR green real-time PCR, each cDNA was amplified with 2× SYBR^®^ supermix (Bio-rad, Hercules, CA, USA) and 3 pmol of each primer. The oligonucleotide primers used for the polymerase chain reaction (PCR) amplification are listed in Table 2.

Real-time PCR reactions were conducted using a CFX96 real-time PCR system (Bio-Rad). PCR amplification was performed under 40 cycles of denaturation at 95 °C for 10 s, followed by 30 s of annealing at 55 °C and 5 s of extension at 95 °C. The relative gene expression C_t_ values were normalized to a *β-actin* endogenous control. 

### 2.9. Statistical Analysis

The results are expressed as the mean value ± standard deviation. Statistical significance was analyzed with one-way analysis of variance (ANOVA) using the Statistical Package for the Social Sciences (SPSS ver. 24.0; IBM, Seoul, Republic of Korea). Differences with *p* values lower than 0.05 were considered statistically significant.

## 3. Results

### 3.1. Chemical Composition of the Essential Oils

The chemical compositions of the essential oils, as determined by GC–MS, are listed in Table 3. Nine compounds, including five monoterpene hydrocarbons and four oxygenated monoterpenes, were identified in all twenty-one citrus cultivars. The most common constituents included α-pinene, D-limonene, trans-β-ocimene, γ-terpinene, terpinolene, linalool, β-terpineol, terpinene-4-ol, and α-terpineol. The essential oils contained high amounts of monoterpene hydrocarbon (83.69–98.95%) with lower amounts of oxygenated sesquiterpene (max. 1.17%). As listed in Table 3, D-limonene (50.88–97.19%) was the main constituent of the essential oils, followed by γ-terpinene (max. 27.29%), β-myrcene (max. 28.09%), α-terpineol (max. 5.85%), and β-pinene (max. 7.55%).

### 3.2. Effects of the Essential Oils on Cell Cytotoxicity

**Figure 1:**
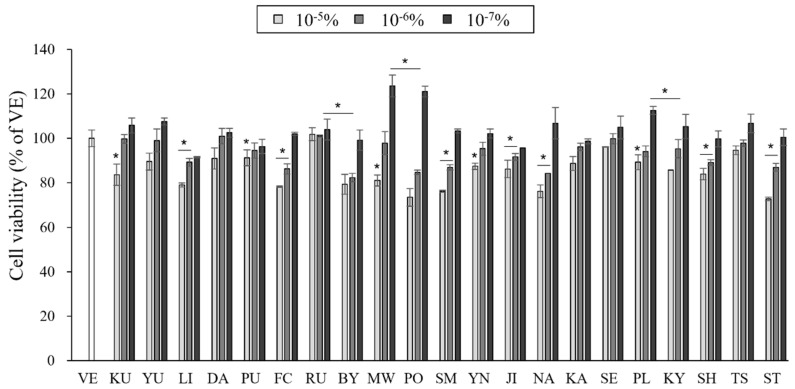
Cell viability measured using a cell counting kit (CCK) assay. Data are presented as mean ± standard deviation. * *p* < 0.05 compared with VE. (BY, *C. platymamma*; DA, *C. maxima*; FC, *C. medica*; JI, *C. sunki*; KA, *C.* X *aurantium*; KY, *C. unshiu* X *C. sinensis*; KU, *C. japonica*; LI, *C. limon*; MW, *C. reticulata*; NA, *C.* X *aurantium*; PL, *C. latifolia*; PO, *C. reticulata*; PU, *C. maxima*; RU, *C. paradisi*; SE, *C.* X *aurantium*; SH, (*C. unshiu* X *C. sinensis*) X *C. reticulata*; SM, *C. reticulata*; ST, ((*C. unshiu* X *C. sinensis*) X *C. reticulata*) X *C. reticulata*; TS, (*C. unshiu* X *C. sinensis*) X *C. unshiu*; YN, *C. sinensis*; YU, *C. junos*).

The cell toxicity of the essential oil samples on RAW 264.7 cells was evaluated to determine adequate concentrations of the essential oil for use. The toxicity of the essential oil samples varied with the dosage (Figure 1).

The cell viability was more than 70% at oil concentrations of 10^−7^–10^−5^%, and almost no cytotoxicity was observed at concentrations lower than 10^−6^%. In some essential oil samples, the cell viability exceeded 100%, and cell proliferation was promoted. Essential oils promote cell proliferation as a defense mechanism against external stimuli [29]. Therefore, to evaluate the anti-inflammatory effects of the essential oils, RAW 264.7 cells were tested with essential oils at a concentration of 10^−6^%.

### 3.3. Effects of the Essential Oils on LPS-Induced NO Production in RAW 264.7 Cells

**Figure 2:**
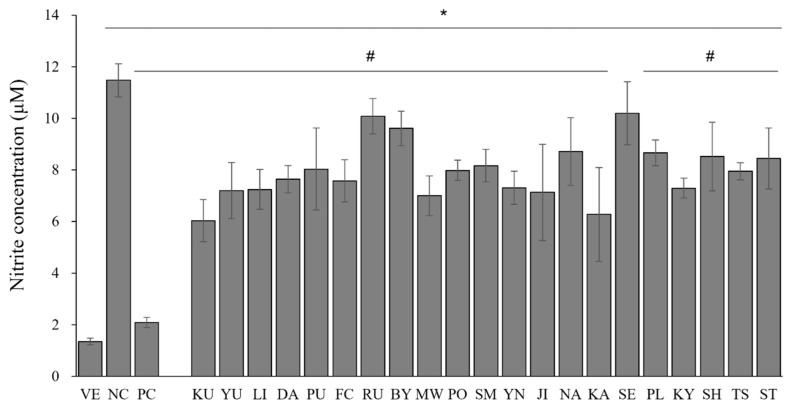
Inhibition effects of essential oils on NO production by LPS-stimulated RAW 264.7 cells. Data are presented as mean ± standard deviation. * *p* < 0.05 compared with VE; # *p* < 0.05 compared with NC. (NC, 1 μg/mL lipopolysaccharide; PC, 100 nM dexamethasone; BY, *C. platymamma*; DA, *C. maxima*; FC, *C. medica*; JI, *C. sunki*; KA, *C.* X *aurantium*; KY, *C. unshiu* X *C. sinensis*; KU, *C. japonica*; LI, *C. limon*; MW, *C. reticulata*; NA, *C.* X *aurantium*; PL, *C. latifolia*; PO, *C. reticulata*; PU, *C. maxima*; RU, *C. paradisi*; SE, *C.* X *aurantium*; SH, (*C. unshiu* X *C. sinensis*) X *C. reticulata*; SM, *C. reticulata*; ST, ((*C. unshiu* X *C. sinensis*) X *C. reticulata*) X *C. reticulata*; TS, (*C. unshiu* X *C. sinensis*) X *C. unshiu*; YN, *C. sinensis*; YU, *C. junos*).

To evaluate the phagocytosis-related activity of the macrophages, the amounts of NO dissolved in the cell supernatants were measured in vitro. The NO concentrations in the LPS-stimulated RAW 264.7 cells are shown in Figure 2. The NO content of the LPS-treated cells (NC) was approximately 8.5-fold higher than that of the untreated cells (VE). The treatment with dexamethasone (100 nM) markedly downregulated the NO content in the positive control by 81.8% compared with that of NC. The essential oil treatment on LPS-stimulated RAW 264.7 cells inhibited NO production by 11.2–47.5% compared to that of NC. For *C. japonica* (KU) and *C.* X *aurantium* (KA), the NO content was reduced by 47.5% and 45.4%, respectively. In addition, the NO content of the essential oils from *C. reticulata* (MW)*, C. sunki* (JI)*,* and *C. junos* (YU) decreased by 39.0%, 37.9%, and 37.3%, respectively.

### 3.4. Effects of Essential Oils on the Gene Expression Levels of Proinflammatory Cytokines

**Figure 3:**
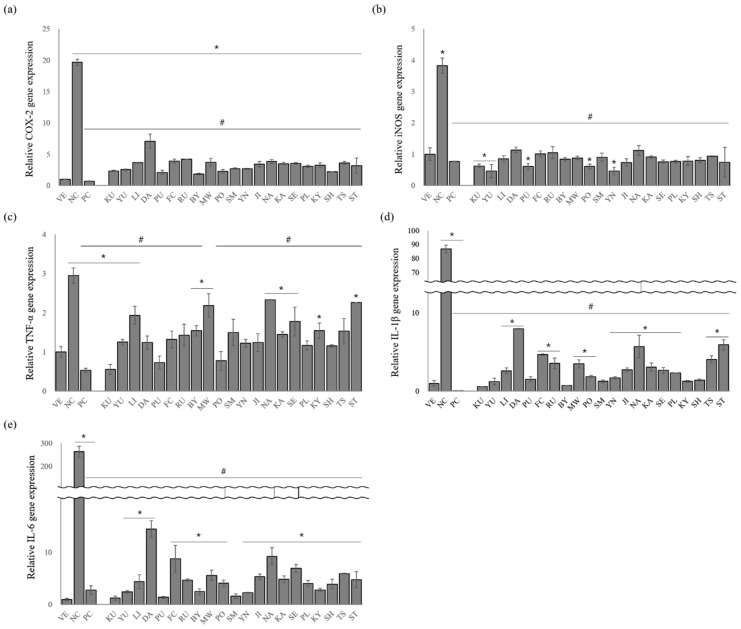
Effect of essential oils on LPS-induced proinflammatory cytokine production: (**a**) *COX-2*, (**b**) *iNOS*, (**c**) *TNF-α*, (**d**) *IL-1β*, and (**e**) *IL-6* expressions. Data are presented as mean ± standard deviation. * *p* < 0.05 compared with VE; # *p* < 0.05 compared with NC. (NC, 1 μg/mL lipopolysaccharide; PC, 100 nM dexamethasone; BY, *C. platymamma*; DA, *C. maxima*; FC, *C. medica*; JI, *C. sunki*; KA, *C.* X *aurantium*; KY, *C. unshiu* X *C. sinensis*; KU, *C. japonica*; LI, *C. limon*; MW, *C. reticulata*; NA, *C.* X *aurantium*; PL, *C. latifolia*; PO, *C. reticulata*; PU, *C. maxima*; RU, *C. paradisi*; SE, *C.* X *aurantium*; SH, (*C. unshiu* X *C. sinensis*) X *C. reticulata*; SM, *C. reticulata*; ST, ((*C. unshiu* X *C. sinensis*) X *C. reticulata*) X *C. reticulata*; TS, (*C. unshiu* X *C. sinensis*) X *C. unshiu*; YN, *C. sinensis*; YU, *C. junos*).

In macrophages, *COX-2*, *iNOS*, *TNF-α*, *IL-1β*, and *IL-6* are the key cytokines involved in the inflammatory responses [30]. Therefore, these cytokines are used as important markers of anti-inflammatory activity. Herein, the extracted essential oils significantly inhibited the production of *COX-2*, *iNOS*, *TNF-α*, *IL-1β*, and *IL-6* expression in LPS-stimulated RAW 264.7 cells (Figure 3).

After treating the RAW 264.7 cells with LPS, the relative *COX-2* expression increased 19.6-fold, whereas dexamethasone treatment decreased the *COX-2* expression by approximately 96.6% (Figure 3a). *C. platymamma* (BY), *C. maxima* (PU), (*C. unshiu* X *C. sinensis*) X *C. reticulata* (SH), *C. reticulata* (PO), and *C. japonica* (KU) remarkably inhibited *COX-2* release by 90.6%, 89.3%, 88.9%, 88.6%, and 88.1%, respectively.

Stimulating the RAW 264.7 cells with LPS (NC) significantly increased the relative expression level of *iNOS* by approximately 3.8-fold compared with that of the VE, whereas *iNOS* expression was suppressed in LPS-stimulated RAW 264.7 cells treated with dexamethasone (79.9%) (Figure 3b). All the oil samples also inhibited *iNOS* secretion from the LPS-stimulated RAW 264.7 cells, and *C. junos* (YU, 87.9%), *C. sinensis* (YN, 87.8%), *C. maxima* (PU, 84.2%), *C. reticulata* (PO, 84.1%), and *C. japonica* (KU, 83.6%) showed the highest inhibitory effects. These inhibition percentages are higher than those observed under dexamethasone treatment.

LPS-stimulated RAW 264.7 cells increased *TNF-α* expression by approximately 2.9-fold compared with that of the VE (Figure 3c). However, this increase in *TNF-α* expression was suppressed by dexamethasone (81.9%). The anti-inflammatory activity of the essential oils showed 20.8–81.3% inhibition compared with that of NC. Among them, oils from *C. japonica* (KU, 81.3%)*, C. maxima* (PU, 75.2%)*,* and *C. reticulata* (PO, 73.7%) showed a higher inhibitory effect on *TNF-α* expression, and the anti-inflammatory activity of *C. japonica* was similar to that of PC.

A remarkable increase in *IL-1β* expression compared with the vehicle (VE) was observed when the RAW 264.7 cells were treated with LPS (Figure 3d). However, IL-1β expression was attenuated by 99.9% after the cells were treated with dexamethasone. IL-1β expression in citrus-oil-treated groups was also suppressed by 90.8–99.3%. Oils from *C. japonica* (KU, 99.3%), *C. platymamma* (BY, 99.1%), *C. unshiu* X *C. sinensis* (YN, 98.6%), and *C. reticulata* (SM, 98.6%) showed higher inhibitory effects on *IL-1β* expression than other species.

LPS-stimulated RAW 264.7 cells had 297-fold-increased *IL-6* expression compared with the untreated group (Figure 3e). The essential oils significantly suppressed *IL-6* release in the LPS-stimulated RAW 264.7 cells compared with the control (NC), and oils from *C. japonica* (KU), *C. maxima* (PU), and *C. reticulata* (SM) were the most effective. *C. japonica* oil effectively inhibited *IL-6* expression (99.5%), and that of *C. maxima* and *C. reticulata* showed inhibitory effects of 99.5% and 99.4%, respectively.

Compared with other essential oil constituents, the essential oil constituents of *C. japonica* (KU) and *C. maxima* (PU) were distinguished into seven distinct constituents, α-pinene, myrcene, D-limonene, β-ocimene, linalool, linalool oxide, and α-terpineol, when statistical analysis was performed. Therefore, it has been suggested that these compounds affect the anti-inflammatory activity of *C. japonica* and *C. maxima* oils. Based on the aforementioned results, the anti-inflammatory activities were evaluated for seven single compounds to identify active anti-inflammatory constituents.

### 3.5. Cytotoxicity of the Single Compounds

**Figure 4:**
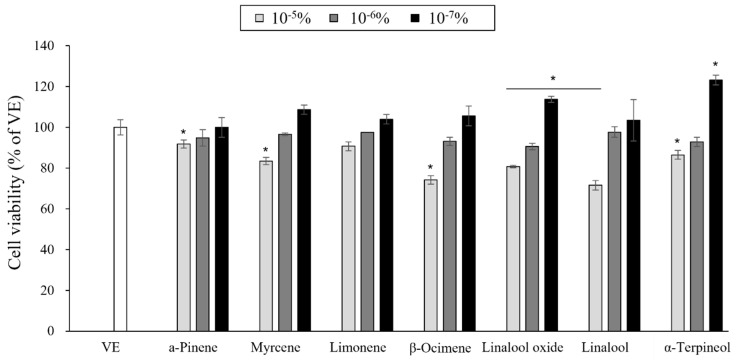
Cell viability of RAW 264.7. The results are presented as mean ± standard deviations of three independent experiments. * *p* < 0.05 compared with VE.

A CCK assay was performed to investigate the cytotoxicity of seven single compounds against RAW 264.7 cells (Figure 4). When the cell viability was higher than 80%, the compounds were considered noncytotoxic and adequate for further analysis. Based on this, the compounds were noncytotoxic at concentrations of 10^−7^–10^−6^%). Therefore, a concentration of 10^−6^% was adopted for subsequent anti-inflammation investigations.

### 3.6. Effects of Single Compounds on LPS-Induced NO in RAW 264.7 Cells

**Figure 5:**
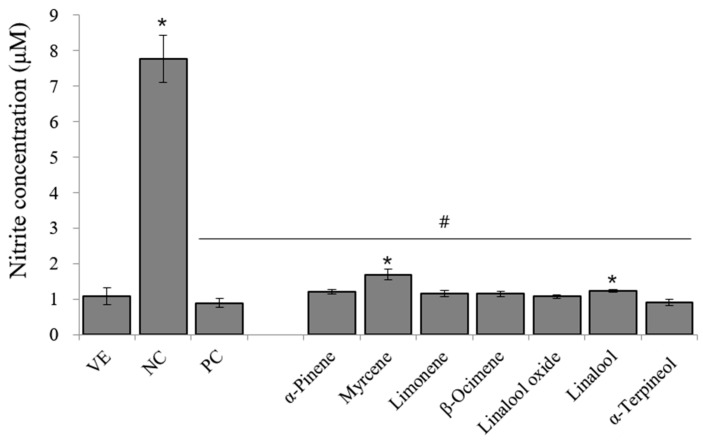
Inhibitory effects of single compounds on NO production. Data are presented as mean ± standard deviations. * *p* < 0.05 compared with VE; # *p* < 0.05 compared to NC.

Based on cell cytotoxicity, a concentration of 10^−6^% was employed for the NO assay. The inhibitory effects of the seven single compounds on NO production by stimulated RAW 264.7 cells are shown in Figure 5. NO production in LPS-stimulated RAW 264.7 cells increased by approximately 7.8-fold compared with that in NC. Dexamethasone inhibited NO production by 88.4%. The seven single compounds significantly inhibited NO production. Among the single compounds, α-terpineol had the highest inhibitory effect on NO production.

### 3.7. Effects of the Single Compounds on the Gene Expression Levels of Proinflammatory Cytokines

**Figure 6:**
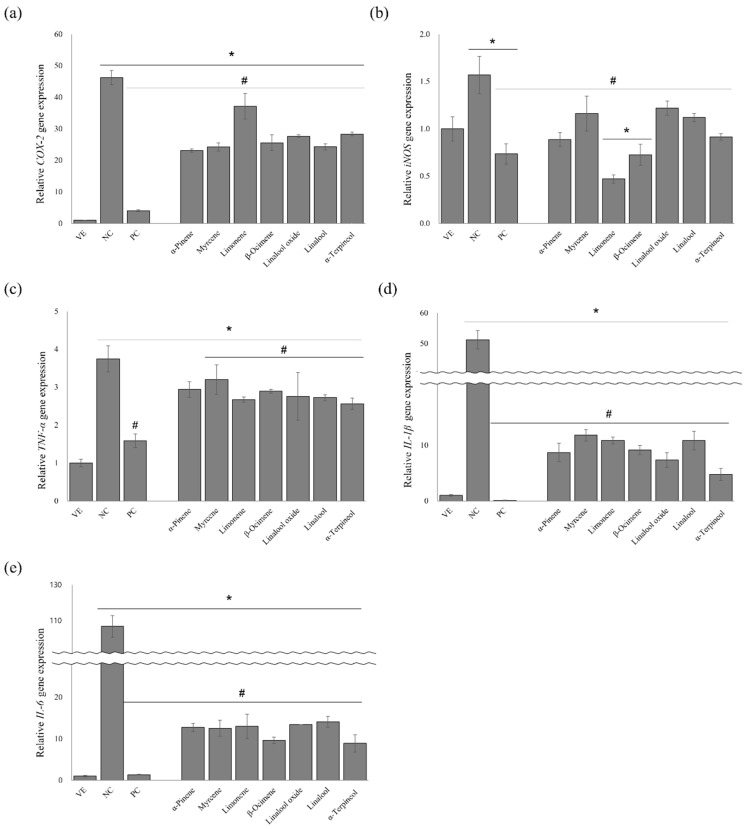
Effect of essential oils on LPS-induced proinflammatory cytokine production: (**a**) *COX-2*, (**b**) *iNOS*, (**c**) *TNF-α*, (**d**) *IL-1β*, and (**e**) *IL-6* expressions. Data presented as mean ± standard deviation. * *p* < 0.05 compared with VE; # *p* < 0.05 compared with NC. (NC, 1 μg/mL lipopolysaccharide; PC, 100 nM dexamethasone).

The anti-inflammatory effects of the seven single compounds were evaluated by measuring *TNF-α*, *iNOS*, *COX-2*, *IL-1β*, and *IL-6* release in the LPS-stimulated RAW 264.7 cells (Figure 6). All the single compounds significantly inhibited *COX-2*, *iNOS*, *TNF-α*, *IL-1β*, and *IL-6* release.

After treating the RAW 264.7 cells with LPS, *COX-2* expression increased 46.3-fold, whereas *COX-2* release was inhibited in the LPS-stimulated RAW 264.7 cells treated with dexamethasone (91.2%) (Figure 6a). The compounds suppressed *COX-2* expression by 19.7–50.1% compared with that of NC. Among them, α-pinene (50.1%) showed the highest inhibitory effect on COX-2 expression.

LPS-stimulated RAW 264.7 cells increased the *iNOS* expression by approximately 1.6-fold compared with that of the VE (Figure 6b). D-Limonene suppressed *iNOS* expression (70.1%) more than that of dexamethasone (53.2%) and other single compounds (below 54%).

Stimulation of RAW 264.7 cells by LPS significantly increased the relative expression level of *TNF-α* 3.8-fold compared with that of the untreated group (Figure 6c). However, the increase in *TNF-a* expression was decreased by dexamethasone treatment (57.8%). α-Terpineol, D-limonene, linalool, linalool oxide, β-ocimene, α-pinene, and myrcene also suppressed *TNF-a* expression by 31.7%, 28.8%, 27.3%, 26.4%, 22.9%, 21.6%, and 14.5%, respectively.

Treating RAW 264.7 cells with LPS remarkably increased *IL-1β* expression by approximately 51.2-fold, whereas dexamethasone treatment decreased *IL-1β* expression by 99.8% (Figure 6d). However, the increase in *IL-1β* expression was suppressed when the cells were treated with the nine following compounds: α-terpineol (90.7%), linalool oxide (85.6%), α-pinene (83.0%), β-ocimene (82.1%), linalool (78.8%), D-limonene (78.8%), and myrcene (77.0%).

LPS treatment significantly increased *IL-6* expression by approximately 106.6-fold, and the increase was significantly suppressed by dexamethasone and the single compounds (Figure 6e). *IL-6* expression in the positive control was suppressed by approximately 98.7% compared with that of NC, and α-terpineol and β-ocimene showed the highest inhibitory effects (91.7% and 91.0%, respectively).

α-Terpineol exhibited an excellent anti-inflammatory effect by suppressing LPS-induced *iNOS* and *COX-2* expressions and the subsequent production of NO in the macrophages. In addition, it inhibited the expression of other proinflammatory cytokines, including *TNF-α*, *IL-1β*, and *IL-6*

## 4. Discussion

Natural products have attracted remarkable attention owing to their few side effects. Specifically, essential oils have been widely investigated as alternative anti-inflammatory reagents. Volatile compounds in essential oils are low-molecular-weight lipophilic compounds that can easily saturate cell membranes. Thus, volatile compounds exhibit anti-inflammatory activity in cells [31]. This study evaluated the anti-inflammatory effects of essential oils from 21 citrus cultivars with various health benefits. To evaluate the anti-inflammatory effect of the essential oils, the levels of inflammatory mediators (NO) and proinflammatory cytokines (*TNF-α*, *COX-2*, *iNOS*, *IL-1β*, and *IL-6*) were investigated.

LPS-induced inflammatory injuries in macrophages are mediated by NO production [32]. Overexpression of the inflammatory mediator (NO) can induce the production of proinflammatory cytokines. Thus, inflammation responses are suppressed by the inhibition of NO production [33]. Herein, the release of proinflammatory mediators was prevented as the amount of NO production was reduced (Figure 2 and Figure 3), revealing the potential anti-inflammatory activities of citrus oils. However, essential oils that strongly inhibited NO production differed from essential oils with superior inhibitory activities of *COX-2* and *iNOS* gene expression. Further studies are still needed to clarify the exact role of the essential oil constituents in the inhibition pathway of NO production and gene expression.

In a previous study, the anti-inflammatory effect of the essential oil from *C. medica* was investigated [27]. The LPS concentrations and the collection sites were the same in both studies, but the extraction parts (fruits and peels) for the essential oils were different. The treatment concentration of the essential oil from the peels was lower, but the activity was higher. This difference is attributed to the chemical compositions and the proportions of the constituents. Even for essential oil derived from the same cultivars, the chemical composition depends on the environmental conditions, origin, and plant part [34].

Among the chemical composition of the 21 citrus oils, the seven single compounds were considered contributing compounds to anti-inflammatory activity. The single compounds significantly inhibited the levels of inflammation-related factors (Figure 5 and Figure 6). Previous studies have shown that the lipophilicity of monoterpenes is promising for regulating inflammatory cytokines owing to their characteristic absorption and rapid response [35]. Monoterpenes decrease inflammatory responses and modulate the key chemical mediators of inflammation. Previous studies reported that monoterpenes, such as borneol, citral, and geraniol, exhibit anti-inflammatory activity by suppressing the LPS-induced production of proinflammatory cytokines and NO [36]. The active anti-inflammatory constituents identified in this and previous studies have structural hydroxyl groups. A study conducted by Ueda et al. indicated that the anti-inflammatory effects of many natural compounds are due to to a hydroxyl group in their structure [37]. However, the exact mechanism of the effect of the hydroxyl group on the anti-inflammatory activity was not elucidated; therefore, Ueda et al. focused on determining how the hydroxyl group affects physiological activity in their study [37].

α-Terpineol has been identified as an active anti-inflammatory compound contributing to the anti-inflammatory activity of *C. japonica* and *C. maxima*. Although the anti-inflammatory activity of *C. japonica* oil was superior to that of the other cultivars, α-terpineol contained less than others. Considering that all seven evaluated compounds exhibited superior anti-inflammatory activity, the anti-inflammatory activity of essential oils is attributed to the synergistic effect of the oil constituents. In *C. japonica* oil, seven single compounds accounted for the highest portion of constitutes present at 99.36% among the 21 citrus cultivars. In a previous study, D-limonene exhibited antimicrobial and anti-yeast effects in synergy with 1,8-cineole and α-pinene [38,39]. However, the synergistic effect of essential oil and the mechanisms involved are not entirely clear. The synergistic potential is difficult to predict, requiring an in-depth knowledge of essential oils, chemical compositions, interactions between constituents, and the action mechanism [40].

It is known that enantiomers exist in terpene compounds, and there is a bioactivity difference between isomers. For instance, the enantiomers of limonene have shown different anti-inflammatory activities: (−)-limonene exhibited approximately three-fold higher anti-inflammatory activity than the (+)-limonene enantiomer [41]. These results are important because the pharmacological activity of essential oils varies with specific enantiomers and/or the ratio of the enantiomers [42]. Therefore, the anti-inflammatory effect of the enantiomers of the single compounds used in this study should also be evaluated in future studies.

## 5. Conclusions

Citrus fruits are globally grown over 11.42 million ha with a production of 179.0 million tons [43]. There are various citrus cultivars, but research has been restricted to only a few cultivars. This study was performed to further increase the utilization of citrus cultivars by studying anti-inflammatory activities of 21 citrus cultivars. The results suggest that *C. japonica* and *C. maxima* are promising candidates for alleviating inflammatory diseases. These research results serve as a scientific basis for the use of essential oils from citrus cultivars in reducing inflammatory symptoms. Components in essential oil must be standardized before citrus oil can be utilized as an inflammatory disease reliever, as a basic step to producing a drug with a consistent and uniform composition. In addition, further research on synergistic effects between potential essential oil components is essential to isolate the active components from essential oils and subsequently use them as single compounds in drug formulations.

## Figures and Tables

**Table 1:** Information about the 21 citrus cultivars [28].

No.	Species	Abbreviation	Specimen Information	Extraction Yield (mL/100 g, Dry Basis)
1	*Citrus japonica* Thunb.	KU	WTFRC10032742	9.52 ± 0.33
2	*Citrus junos* Siebold ex Tanaka	YU	WTFRC10032743	6.93 ± 1.04
3	*Citrus limon* (L.) Osbeck ‘Lisbon’	LI	WTFRC10033803	5.05 ± 1.13
4	*Citrus maxima* (Burm.) Merr.	DA	WTFRC10032725	6.75 ± 1.41
5	*Citrus maxima* (Burm.) Merr. ^a^	PU	WTFRC10032744	2.07 ± 0.60
6	*Citrus medica* L. ^b^	FC	WTFRC10033804	10.09 ± 0.01
7	*Citrus paradisi* Macfad. ‘Redblush’	RU	WTFRC10032741	3.70 ± 0.84
8	*Citrus platymamma* hort. ex Tanaka	BY	WTFRC10032726	5.93 ± 0.22
9	*Citrus reticulata* Blanco ^c^	MW	WTFRC10032727	3.59 ± 1.00
10	*Citrus reticulata* Blanco ‘Ponkan’	PO	WTFRC10032734	16.25 ± 0.01
11	*Citrus reticulata* Blanco ^d^	SM	WTFRC10032740	4.85 ± 0.14
12	*Citrus sinensis* (L.) Osbeck ‘Navel’	YN	WTFRC10032732	6.39 ± 0.30
13	*Citrus sunki* (Hayata) Yu. Tanaka	JI	WTFRC10032733	6.22 ± 0.69
14	*Citrus* X *aurantium* L. ^e^	NA	WTFRC10032737	2.03 ± 0.36
15	*Citrus* X *aurantium* L. ^f^	KA	WTFRC10032735	10.25 ± 0.40
16	*Citrus* X *aurantium* L. ^g^	SE	WTFRC10032729	10.43 ± 0.01
17	*Citrus* X *latifolia* (Yu. Tanaka) Yu. Tanaka	PL	WTFRC10032736	5.04 ± 0.01
18	*Citrus unshiu* X *C. sinensis*	KY	WTFRC10032739	5.49 ± 0.39
19	(*Citrus unshiu* X *C. sinensis*) X *C. reticulata*	SH	WTFRC10032728	6.47 ± 0.18
20	(*Citrus unshiu* X *C. sinensis*) X *C. unshiu*	TS	WTFRC10032731	6.05 ± 1.35
21	((*Citrus unshiu* X *C. sinensis*) X *C. reticulata*) X *C. reticulata*	ST	WTFRC10032730	6.96 ± 0.48

^a^ Synonym of *C. grandis* (L.) Osbeck, ^b^ synonym of *C. medica* L. var. *sarcodactylus* (Siebold ex Hoola van Nooten) Swingle, ^c^ synonym of *C. unshiu* (Yu.Tanaka ex Swingle) Marcow. ‘Miyagawa-wase’, ^d^ synonym of *C. unshiu* (Yu.Tanaka ex Swingle) Marcow., ^e^ synonym of *C.* X *natsudaidai* (Yu.Tanaka) Hayata, ^f^ synonym of *C.* X *benikoji* Yu.Tanaka, ^g^ synonym of *C.* X *tangelo* J.W.Ingram & H.E.Moore.

**Table 2:** Oligonucleotide primer sequences used for the quantitative real-time polymerase chain reaction.

Gene	Primer Sequence (5′–3′)
*IL-1β*	F: CAG GAT GAG GAC ATG AGC ACC R: CTC TGC AGA CTC AAA CTC CAC
*IL-6*	F: GTA CTC CAG AAG ACC AGA GG R: TGC TGG TGA CAA CCA CGG CC
*COX-2*	F: CGG ACT GGA TTC TAT GGT GAA A R: CTT GAA GTG GGT CAG GAT GTA G
*iNOS*	F: CCC TTC CGA AGT TTC TGG CAG CAG C R: GGC TGT CAG AGC CTC GTG GCT TTG G
*TNF-α*	F: TCC AGG CGG TGC CTA TGT R: CGA TCA CCC CGA AGT TCA GT
*β-actin*	F: CAG GTC ATC ACT ATT GGC AA R: AGG TCT TTA CGG ATG TCA AC

**Table 3:** Chemical composition of essential oil from citrus cultivar peels.

KI ^a^	Compound Name	Area %																				
		KU	YU	LI	DA	PU	FC	RU	BY	MW	PO	SM	YN	JI	NA	KA	SE	PL	KY	SH	TS	ST
Monoterpene Hydrocarbons																						
920	α-Thujene	-	0.17	0.12	-	-	0.64	-	-	0.06	0.09	0.09	-	-	0.03	-	0.02	0.19	-	-	0.07	-
926	α-Pinene	0.32	1.06	1.05	0.26	0.19	2.00	0.38	0.30	0.61	0.68	0.67	0.34	0.45	0.46	0.41	0.55	1.64	0.32	0.42	0.61	0.49
939	α-Fenchene	-	-	-	-	-	-	-	-	-	-	-	-	-	-	-	-	0.02	-	-	-	-
941	Camphene	-	-	0.05	-	-	-	-	-	-	-	-	-	-	-	-	-	0.09	-	-	-	-
965	Sabinene	-	0.01	0.13	-	-	0.07	0.29	0.05	0.01	0.08	0.02	0.04	0.11	-	0.03	-	0.21	0.03	0.16	0.20	0.63
970	β-Pinene	-	0.50	6.03	-	0.22	1.62	0.14	0.85	0.25	0.26	0.34	-	1.98	0.21	0.86	0.12	7.55	-	0.04	0.27	0.36
986	β-Myrcene	1.34	1.25	0.95	21.62	28.09	-	1.25	19.48	1.21	1.17	1.27	1.31	1.17	1.09	1.23	1.31	0.84	1.23	1.36	1.18	1.05
1006	Cosmene	-	-	-	-	-	-	-	-	-	-	-	-	-	-	-	-	-	-	-	-	0.03
1008	α-Phellandrene	0.01	0.49	0.05	0.02	0.03	0.04	0.05	0.02	0.02	0.03	0.02	0.04	-	0.04	0.02	0.02	0.07	0.01	0.04	0.03	0.02
1012	3-Carene	-	-	-	-	-	-	-	-	-	-	-	0.07	-	-	-	-	-	-	-	-	-
1024	α-Terpinene	-	0.39	0.34	-	-	0.52	0.28	0.02	0.09	0.11	0.11	0.07	0.05	0.10	0.02	0.07	0.60	0.05	0.17	0.18	0.42
1034	m-Cymene	-	0.63	1.33	-	0.04	0.83	0.04	-	0.27	0.13	0.39	0.01	-	0.21	-	0.10	1.90	-	-	1.29	0.05
1041	D-Limonene	97.19	77.98	69.01	76.33	68.79	59.19	93.47	77.13	90.59	90.50	89.32	95.74	92.35	90.40	95.19	91.96	50.88	96.49	94.24	89.21	91.11
1047	cis-β-Ocimene	-	-	0.04	0.06	-	1.16	-	0.13	-	-	-	-	0.10	-	0.01	-	0.04	-	0.10	-	-
1058	trans-β-Ocimene	0.04	0.31	0.10	0.40	0.20	1.76	0.44	0.52	0.07	0.06	0.10	0.04	0.38	0.19	0.10	0.32	0.10	0.07	0.44	0.20	0.14
1068	γ-Terpinene	0.01	11.53	9.73	0.02	0.08	27.29	0.65	0.07	4.57	5.05	5.49	0.17	0.11	5.03	0.06	3.42	17.61	0.12	0.37	3.89	0.84
1090	Terpinolene	0.04	0.70	1.01	0.14	0.21	1.25	0.31	0.06	0.26	0.26	0.33	0.09	0.11	0.37	0.19	0.24	1.92	0.06	0.12	0.27	0.23
1093	ρ,α-Dimethylstyrene	-	0.03	0.01	-	-	-	-	-	-	-	0.01	-	-	-	-	-	0.03	-	-	-	0.04
Oxygenated Monoterpene																						
1004	Octanal	-	-	0.02	-	-	-	-	-	-	0.08	-	0.09	-	0.04	-	-	-	0.10	0.04	0.20	-
1042	1,8-Cineole	-	-	-	-	-	-	-	-	-	-	-	-	-	-	-	-	0.25	-	-	-	-
1078	Linalool oxide	0.02	0.04	-	0.25	0.28	-	0.24	-	-	-	0.01	-	-	0.11	0.18	-	-	-	-	0.02	-
1099	Linalool	0.14	1.97	0.37	0.10	0.18	0.06	0.23	0.42	0.42	0.71	0.12	0.35	0.73	0.17	0.57	0.33	0.72	0.11	0.45	0.29	0.95
1104	Nonanal	-	-	0.08	-	-	-	-	-	0.01	-	-	-	-	0.01	-	-	-	0.04	0.01	0.07	-
1116	D-Fenchyl alcohol	-	-	0.10	-	-	-	-	-	-	-	-	-	0.01	-	-	-	0.19	-	-	-	-
1119	trans-ρ-2,8,1-Menthadienol	-	-	-	-	0.06	-	-	-	-	-	-	-	-	-	-	-	-	-	-	-	0.07
1122	(Z)-ρ-2-Menthen-1-ol	-	0.02	-	-	-	-	-	-	-	-	-	-	-	-	-	-	-	-	0.01	0.01	0.04
1146	β-Terpineol	0.05	0.10	0.11	0.03	0.06	0.01	0.07	0.02	0.05	0.02	0.03	0.12	0.03	0.12	0.09	0.17	0.13	0.08	0.10	0.04	0.06
1149	Citronella	-	-	-	-	-	0.01	-	-	-	-	-	-	-	-	0.01	-	-	-	0.03	-	-
1168	α-Phellandren-8-ol	-	-	-	-	-	-	-	-	-	-	-	-	-	-	-	-	0.19	-	-	-	-
1169	Borneol	-	-	0.17	-	-	-	-	-	-	-	-	-	-	-	-	-	0.16	-	-	-	-
1179	Terpinen-4-ol	0.03	0.34	1.19	0.04	0.08	0.46	1.14	0.13	0.11	0.17	0.11	0.31	0.24	0.15	0.13	0.13	1.69	0.21	0.71	0.66	1.78
1186	ρ-Cymen-8-ol	-	-	-	-	-	-	-	-	-	-	-	-	-	-	-	-	0.02	-	-	-	-
1194	α-Terpineol	0.28	1.01	3.28	0.19	0.37	0.66	0.57	0.28	0.37	0.26	0.26	0.58	0.49	0.77	0.58	0.96	5.85	0.45	0.51	0.41	0.51
1206	Decanal	-	-	0.02	-	-	-	0.11	-	0.07	0.08	0.01	0.10	-	0.07	-	-	0.08	0.10	0.32	0.31	0.20
1222	trans-Carveol	-	-	-	-	-	-	-	-	-	-	-	-	-	-	-	-	-	-	-	-	0.24
1228	Nerol	-	-	0.08	-	-	-	-	-	-	-	-	-	-	-	-	-	-	-	-	-	-
1238	cis-Carveol	-	-	-	-	-	-	-	-	-	-	-	-	-	-	-	-	-	-	-	-	0.07
1243	(Z)-Citral	-	-	1.44	-	0.08	0.26	-	-	-	-	-	-	-	-	-	-	1.70	-	-	-	-
1251	Carvone	-	-	-	-	-	-	-	-	-	-	-	-	-	-	-	-	-	-	-	-	0.10
1257	Geraniol	-	-	0.06	-	-	-	-	-	-	-	-	-	-	-	-	-	-	-	-	-	-
1273	(E)-Citral	-	-	2.17	-	0.07	0.26	-	-	-	-	-	-	-	-	-	-	2.43	-	-	-	-
1288	Bornyl acetate	-	-	-	-	-	-	-	-	-	-	-	0.07	-	-	-	-	-	-	-	-	-
Sesquiterpene Hydrocarbons																						
1343	δ-Elemene	0.06	0.07	-	0.01	-	-	-	0.01	0.06	0.02	0.06	-	0.03	-	0.01	-	0.06	-	-	-	-
1384	α-Copaene	0.01	0.01	-	0.01	-	-	0.03	-	0.01	-	0.01	-	0.04	0.01	0.01	0.01	-	-	-	0.01	-
1395	β-Elemene	0.03	0.05	-	0.01	-	-	0.02	0.03	0.49	0.01	0.72	0.01	0.97	0.09	0.05	0.01	0.10	0.02	-	0.14	0.06
1430	Caryophyllene	-	0.13	0.08	-	-	-	-	-	-	-	-	-	-	-	-	-	0.26	-	-	-	-
1437	γ-Elemene	-	0.06	-	-	-	-	-	-	-	-	-	-	-	-	-	-	-	-	-	-	-
1440	trans-α-Bergamotene	-	0.01	0.08	-	0.02	0.13	-	-	-	-	-	-	-	0.03	-	-	0.33	-	-	0.01	-
1457	β-Farnesene	-	0.21	-	-	-	-	0.01	0.03	-	-	-	-	-	-	-	-	-	-	-	0.05	0.01
1467	Humulene	-	0.03	0.01	-	-	0.02	0.03	0.01	0.06	-	0.08	-	0.11	0.01	0.01	0.01	0.05	-	-	0.02	-
1484	α-Selinene	0.01	-	-	-	-	0.01	-	-	-	-	-	-	0.01	-	-	-	-	-	-	-	-
1488	α-Muurolene	0.01	-	-	-	-	-	-	-	-	-	-	-	-	-	-	-	-	-	-	-	-
1491	Germacrene D	0.07	0.03	-	0.11	-	0.14	-	0.03	0.04	0.01	0.05	-	0.03	0.01	0.01	-	0.03	-	-	-	-
1500	β-Selinene	-	0.15	-	-	-	0.01	-	-	-	-	0.01	-	0.02	-	-	-	0.02	-	-	-	-
1502	Valencene	-	-	0.01	-	-	-	-	-	0.01	-	0.01	0.33	-	-	-	0.05	-	0.06	0.01	-	-
1508	α-Farnesene	-	0.11	-	0.01	-	0.03	-	0.04	0.01	-	0.11	0.02	0.04	0.03	-	-	0.07	-	0.11	0.01	-
1513	β-Bisabolene	0.01	-	0.10	-	-	0.14	-	0.06	0.08	-	-	-	-	0.01	-	-	0.53	-	-	-	-
1528	δ-Cadinene	0.03	0.08	-	0.03	-	0.04	0.07	0.01	0.04	-	0.05	0.01	0.09	0.02	0.02	0.02	-	0.01	0.01	-	-
Oxygenated Sesquiterpene																						
1352	α-Terpinyl acetate	-	-	0.01	-	-	-	0.02	-	-	-	-	-	0.05	-	-	-	-	-	-	-	-
1362	Neryl acetate	-	-	0.25	-	-	-	-	-	-	-	-	-	-	0.06	-	0.04	0.89	0.06	-	0.10	-
1379	Geranyl acetate	0.05	-	0.24	-	-	-	-	-	-	-	-	-	-	-	-	-	0.28	-	-	-	-
1586	Veridiflorol	-	0.03	-	-	-	-	-	-	-	-	-	-	-	-	-	-	-	-	-	-	-
1641	β-Eudesmol	-	0.02	-	-	-	-	-	-	-	-	-	-	-	-	-	-	-	-	-	-	-
1661	τ-Muurolol	0.01	0.03	-	-	-	0.01	-	-	0.01	-	0.01	-	0.01	-	-	-	-	-	-	-	-
1674	α-Cadinol	0.02	0.08	-	0.01	-	0.02	-	-	0.02	-	0.01	-	0.02	-	-	-	-	-	-	-	-
Monoterpene hydrocarbons		98.38	98.95	95.05	89.97	98.85	97.84	96.37	97.29	98.63	98.01	98.44	98.16	97.93	96.82	98.15	98.12	98.13	83.69	98.38	97.47	97.41
Oxygenated monoterpenes		1.10	0.52	3.48	9.10	0.61	1.18	1.71	2.36	0.85	1.04	1.33	0.54	1.62	1.51	1.45	1.57	1.60	13.43	1.10	2.18	2.01
Sesquiterpene hydrocarbons		0.09	0.23	0.96	0.29	0.18	0.02	0.53	0.16	0.22	0.81	0.04	1.11	0.37	1.35	0.21	0.11	0.10	1.46	0.09	0.13	0.25
Oxygenated sesquiterpenes		0.06	0.08	0.16	0.49	0.01	-	0.03	0.02	-	0.03	-	0.02	-	0.08	0.06	-	0.04	1.17	0.06	-	0.10
Unknown compounds		0.35	0.21	0.37	0.14	0.37	1.00	0.26	0.15	0.30	0.15	0.17	0.20	0.11	0.25	0.08	0.21	0.13	0.22	0.35	0.23	0.21
Total		100	100	100	100	100	99	99	100	100	100	100	100	100	100	100	100	100	100	100	100	100

^a^ Kovats retention index was experimentally determined using a VF-5MS column with a homologous series of C_8_–C_30_ alkanes. (BY, *C. platymamma*; DA, *C. maxima*; FC, *C. medica*; JI, *C. sunki*; KA, *C.* X *aurantium*; KY, *C. unshiu* X *C. sinensis*; KU, *C. japonica*; LI, *C. limon*; MW, *C. reticulata*; NA, *C.* X *aurantium*; PL, *C. latifolia*; PO, *C. reticulata*; PU, *C. maxima*; RU, *C. paradisi*; SE, *C.* X *aurantium*; SH, (*C. unshiu* X *C. sinensis*) X *C. reticulata*; SM, *C. reticulata*; ST, ((*C. unshiu* X *C. sinensis*) X *C. reticulata*) X *C. reticulata*; TS, (*C. unshiu* X *C. sinensis*) X *C. unshiu*; YN, *C. sinensis*; YU, *C. junos*).

## Data Availability

Not applicable.

## Abbreviations

ANOVA, analysis was performed based on repeated one-way analysis of variance; CCK, cell counting kit; DMEM, Dulbecco’s modified Eagle’s medium; DPBS, Dulbecco’s phosphate-buffered saline; EI, electron ionization; FBS, fetal bovine serum; FID, flame ionization detector; GC, gas chromatographer; KI, Kovats index; MS, mass spectroscopy; PCR, polymerase chain reaction; SPSS, Statistical Package for the Social Sciences.
